# Distinguishing between cancer driver and passenger gene alteration candidates via cross-species comparison: a pilot study

**DOI:** 10.1186/1471-2407-10-426

**Published:** 2010-08-13

**Authors:** Xinglai Ji, Jie Tang, Richard Halberg, Dana Busam, Steve Ferriera, Maria Marjorette O Peña, Chinnambally Venkataramu, Timothy J Yeatman, Shaying Zhao

**Affiliations:** 1 Department of Biochemistry and Molecular Biology, Institute of Bioinformatics, University of Georgia, Athens 30602, GA, USA; 2 McArdle Laboratory for Cancer Research, University of Wisconsin Madison, WI 53703, USA; 3 J. Craig Venter Institute, Rockville, MD 20850, USA; 4 Center for Colon Cancer Research, University of South Carolina, Columbia, SC 29208, USA; 5 Departments of Surgery, Pathology, and Biostatistics, H. Lee Moffitt Cancer Center and Research Institute, Tampa, FL 33612, USA

## Abstract

**Background:**

We are developing a cross-species comparison strategy to distinguish between cancer driver- and passenger gene alteration candidates, by utilizing the difference in genomic location of orthologous genes between the human and other mammals. As an initial test of this strategy, we conducted a pilot study with human colorectal cancer (CRC) and its mouse model C57BL/6J *Apc*^Min/+^, focusing on human 5q22.2 and 18q21.1-q21.2.

**Methods:**

We first performed bioinformatics analysis on the evolution of 5q22.2 and 18q21.1-q21.2 regions. Then, we performed exon-targeted sequencing, real time quantitative polymerase chain reaction (qPCR), and real time quantitative reverse transcriptase PCR (qRT-PCR) analyses on a number of genes of both regions with both human and mouse colon tumors.

**Results:**

These two regions (5q22.2 and 18q21.1-q21.2) are frequently deleted in human CRCs and encode genuine colorectal tumor suppressors *APC *and *SMAD4*. They also encode genes such as *MCC *(*mutated in colorectal cancer*) with their role in CRC etiology unknown. We have discovered that both regions are evolutionarily unstable, resulting in genes that are clustered in each human region being found scattered at several distinct loci in the genome of many other species. For instance, *APC *and *MCC *are within 200 kb apart in human 5q22.2 but are 10 Mb apart in the mouse genome. Importantly, our analyses revealed that, while known CRC driver genes *APC *and *SMAD4 *were disrupted in both human colorectal tumors and tumors from *Apc*^Min/+ ^mice, the questionable *MCC *gene was disrupted in human tumors but appeared to be intact in mouse tumors.

**Conclusions:**

These results indicate that *MCC *may not actually play any causative role in early colorectal tumorigenesis. We also hypothesize that its disruption in human CRCs is likely a mere result of its close proximity to *APC *in the human genome. Expanding this pilot study to the entire genome may identify more questionable genes like *MCC*, facilitating the discovery of new CRC driver gene candidates.

## Background

Cancer is a disease of the genome. As cancer initiates and progresses, genomic instability develops and abnormal genomic changes (e.g., sequence mutations, aberrant promoter methylation, and structural lesions such as gains/losses, inversions and translocations) accumulate [1-6]. While some of these abnormalities disrupt normal cellular processes and contribute to cancer initiation and progression (i.e., drivers), others emerge simply as victims of genomic instability occurring as a result of cancer progression (i.e., passengers). Clearly, finding genomic abnormalities is significant, but identifying those that are cancer-drivers is even more meaningful.

A central aim of cancer research has been to identify driver gene alterations. This has become both urgent and increasingly challenging in recent years with the advance of sequencing and other technologies [7] as well as the launch of high-throughput cancer genome projects, such as the Cancer Genome Atlas (cancergenome.nih.gov), the International Cancer Genome Consortium http://www.icgc.org, and others [3,4]. Researchers have been tackling this challenge by improving experimental conditions [5] and developing more sophisticated statistical models and functional analysis strategies [3,4,6].

We are developing a cross-species comparison strategy that differs fundamentally from the current published approaches described above (which study human cancers only). We hypothesize that driver alteration candidates can be distinguished from passenger candidates through examination of orthologous genes or genomic loci with tumors from multiple species having the same type of cancer. Provided that these species share similar molecular and genetic pathways of cancer development and progression, abnormalities that are recurrent among different species will have a higher probability to be drivers, whereas those that are found in only one species and are located in evolutionarily unstable sites will be more likely to be passengers. In our studies, evolutionarily unstable sites are defined as regions enriched with interspecies genomic rearrangement breakpoints [8,9]. However, we do acknowledge that our strategy will not be able to identify species-specific drivers.

Our strategy rests on the same rationale that cancer researchers have been using for years: abnormalities recurrent among different cases are more likely to be drivers, compared with non-recurrent events. The difference is that we are searching for events that are recurrent not only among different cases within the same species, but also among different species. One significant advantage of our cross-species comparison strategy has over single-species approaches is that it utilizes the difference in the genomic location of orthologous genes and loci, caused by interspecies genomic rearrangements that occurred during evolution, for driver and passenger alteration distinction. The pilot study illustrating this is described here, which compared human colorectal cancer (CRC) and one of its mouse models C57BL/6J (B6) *Apc*^Min/+ ^[10,11] on genes encoded in two evolutionarily unstable human genomic regions 5q22.2 and 18q21.1-q21.2.

**Figure 1:**
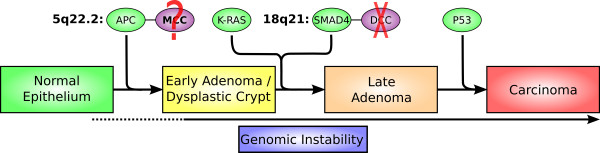
**The human colorectal tumorigenesis model proposed by Vogelstein and colleagues **[12,13]** describes sequential inactivation of tumor suppressors (*APC, SMAD4*, and *P53*), activation of oncogene *KRAS*, and development of genomic instability**. However, genes that are near the bona fide tumor suppressors and are disrupted in the human CRC appear not to be cancer-drivers, based on mouse model studies (*DCC*) or due to lack of evidence for the contribution to cancer (*MCC*).

Human CRC is one of the better understood systems for studying the genetics of cancer initiation and progression [12-17]. The stepwise model of human CRC development and progression proposed by Vogelstein and colleagues [12,13] (Figure 1) includes: 1) inactivation of the *APC, SMAD4*, and *P53 *tumor suppressors; 2) overactivation of the *KRAS *oncogene; and 3) development of genomic instability in the form of either chromosomal instability (CIN) [12-16] or microsatellite instability (MSI) [16,17].

The human 5q22.2 and 18q21.1-q21.2 regions are both constantly disrupted in human CRCs [15,16], and encode bona fide CRC driver genes (e.g., *APC*, *SMAD4*) as well as genes whose role in cancer etiology remains unclear (e.g., *mutated in colorectal cancer or MCC*). By comparison with different species, we found that both regions are evolutionarily unstable and prone to interspecies genomic rearrangements during evolution. As a consequence, genes clustered in each human region were, by contrast, found scattered at several different loci in the genome of many other species, including the mouse. We investigated these genes in colon tumors from humans as well as a well-known CRC mouse model B6 *Apc*^Min/+ ^[10,11]. We found that, while bona fide driver genes *APC *and *SMAD4 *were indeed disrupted in both species (another CRC gene *P53*, located in a different site 17p, was also altered), the questionable *MCC *was altered in human tumors but appeared to be structurally and expressionally intact in mouse tumors. Our results indicate that *MCC *is unlikely a player in early colorectal tumorigenesis, and we hypothesize that its disruption in human colorectal tumors is most likely a mere result of its close proximity to *APC *in the human genome. This study has demonstrated the promise of using this cross-species comparative genomics and oncology strategy for distinguishing between CRC driver- and passenger gene alteration candidates.

## Methods

### Materials

Human DNA samples purified from 40 normal/tumor paired tissues of sporadic Dukes B tumors were provided by Dr. Timothy J. Yeatman from H. Lee Moffitt Cancer Center and Research Institute in Florida. Dr. Yeatman has been approved for human subject use by Institutional Review Boards of the University of South Florida. Colonic adenomas and matching normal tissues from several B6 *Apc*^Min/+ ^mice were provided by Dr. Richard Halberg of the University of Wisconsin (UW) in Madison and Dr. Maria Marjorette O. Peña of the Mouse Core Facility of the Center for Colon Cancer Research of the University of South Carolina (USC). All animal studies were conducted under protocols approved by the Institutional Animal Care and Use Committee of UW and USC, following the guidelines of the American Association for the Assessment and Accreditation of Laboratory Animal Care. Genomic DNA samples were extracted from these tissues using the QIAGEN DNeasy Tissue kit.

### Comparative genomics and sequence analyses

The genomic sequence and annotation data of human 5q22.2 and 18q21.1-q21.2 as well as their homologues from other species were downloaded from the Ensembl site at http://www.ensembl.org and the University of California Santa Cruz genome site at http://www.genome.ucsc.edu. Sequences of different species were aligned through comparison with the BLATN program and/or by examining the gene order of each species. Evolutionary breakpoints were identified by examining the alignments. Repetitive sequences were identified by using the RepeatMasker program obtained from http://www.repeatmasker.org.

### Bi-directional exon resequencing and mutation detection

Sequencing was performed at The J. Craig Venter Institute in Maryland following the established protocols. Briefly, primers were designed based on genomic sequences obtained from the Ensembl site, flanking each exon or within the exon (for big exons) with ~400-800 bp distance apart. Then, the targeted regions were amplified by Hot-Start PCR and the PCR products were cleaned up using Shrimp Alkaline Phosphatase/Exonuclease I mix. Each PCR amplicon was sequenced from both forward and reverse directions, using the Big Dye Terminator chemistry and the ABI 3730*xl *platform.

#### Sequence trimming

Phred, a base call program obtained from Dr. Brent Ewing at The University of Washington http://www.phrap.org/phredphrapconsed.html, was used to obtain the DNA base sequences and the associated quality scores from each sequence trace. Then, lucy, a sequence cleaning program obtained from the J. Craig Venter Institute http://www.jcvi.org/cms/research/software/, was used to trim off low quality bases from each end of the sequences.

#### Mutation detection

Both forward and reverse sequences of each amplicon were used to assemble the final sequence for each exon. The assembled sequences from the tumor/normal paired samples were aligned using the multiple sequence alignment program CLUSTALW, and each alignment was manually inspected to ensure its correctness. Sequence changes (e.g. indels, base substitutions) were identified by finding base changes between each tumor and its matching normal sequences. A cut off phred quality score of 20 was used to reduce false positives due to sequencing errors, ensuring that only high quality bases with an error rate of ≤ 1% were eligible for mutation findings.

### qRT-PCR with mouse genes

#### Primer design

The primers were designed with Primer3 (frodo.wi.mit.edu/primer3/), with the criteria of 20 ± 1 mer primers, Tm of 60 ± 1°C, < 2 G or C residues in the final 5 bases, an amplicon size of 65-75bp, and unique sequences when compared to the RefSeq-RNA database from GenBank and the genomic sequences of the species involved. The primers were "TCTCTCCAAGCAGCGAGAAT" and "ACTTGGACGCAGCTGATTCT" for *APC*; "AGAAGATAGACCGCCTGCAA" and "GCTGAGCTCTGACCGAAGTT" for *MCC*; "GATCGGTGGCTCCATCCTGG" and "GCCGGACTCATCGTACTCCTG" for *β-actin*; and "AGCGAGCGACCAAAGGAACC" and "GCATGTCTAAGTACGCACGGC" for *18 S Ribosomal subunit*. The primer sequences are all indicated in the 5'- to 3'- direction.

#### RNA extraction

The total RNA was extracted using the STAT-60 reagent from Tel-Test (Texas), following the manufacture's instruction. Then, the amount of RNA was determined spectrophotometrically, and only samples with a 260/280 ratio of ~1.8 were subjected to further analyses described below.

#### cDNA synthesis

For each sample, 6 μg RNA was treated with DNase to degrade any residual genomic DNA in the RNA samples using the TURBO DNA-*free*™ kit from Ambion (Texas). Then, 1 μg RNA was used for cDNA synthesis with the RETROscript kit from Ambion, following the manufacture's instructions.

#### qRT-PCR reactions

qRT-PCR reactions were performed in triplicates with each well containing 10 μl iQ™ SYBER Green Supermix from Bio-Rad, 500 nM primers each, and 0.25 μl cDNA synthesized above in a total reaction volume of 20 μl, with an iCycler iQ Real-Time PCR machine. The amplification condition was: 95°C for 10 seconds, 65°C for 45 seconds, and 78°C for 20 seconds for a total of 40 cycles.

### qPCR with mouse genes

The same protocol described for qRT-PCR above was followed except that 10 ng genomic DNA was used in each well instead of cDNA. The primers flanking *SMAD*4 exon 8 (CDS7) were "tttcttcttagGGCCAGTTCAC" and "tacCAGGATGATTGGAAATGG"; and for exon 4 were "CCCACTGAAGGACATTCGAT" and "CTGTACGTCTCCGTTGATGC". The primers of reference controls were "TCCAGGTAGCAATGACGAGA" and "GCGCAGAGCTTGTGTGTAAA" for *MBD2 *exon5; "AGAACTGGTCAGTGCCTTGG", and "TCT GCTGACTGCTGGTGTCT" for *MCC *exon 16; "AGCAGGATTGCGTCCATATC" and "CAGTTGGAGTTGTCGAGCAG" for *MBD1 *exon 10; and "GATGTGCCTATGGTCCTGGT" and "CCTGAGCCTGTTTCGTGTCT" for *KRAS *exon 4.

### Mouse tumor aCGH analysis

The oligo arrays were performed using the 385 K oligonucleotide arrays from Roche NimbeGen, with each chip containing 385,000 oligo probes of ~50 bp unique sequences selected across the mouse genome, providing a resolution of 1 probe every 5 kb on average. Amplifications and deletions were identified by our newly developed software tool SEG (manuscript submitted).

## Results

### Human 5q22.2 and 18q21.1-q21.2 are evolutionarily unstable

**Figure 2:**
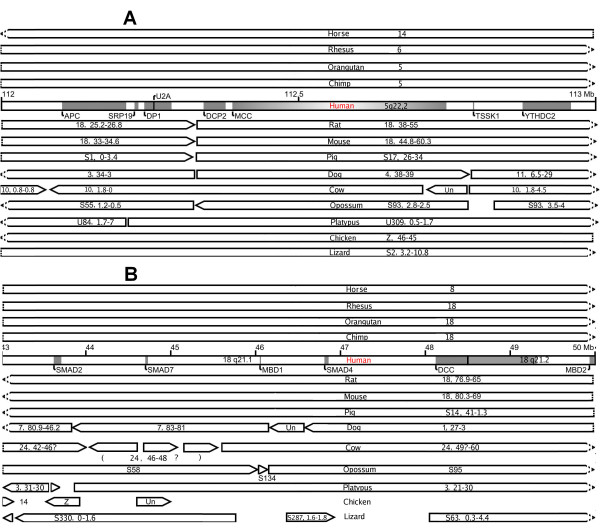
**Human 5q22.2 and 18q21.1-21.2 are evolutionarily unstable**. **A**: Human 5q22.2 (112-113 Mb) is shown as the bar with its gene-coding regions (*APC, MCC*, etc.) shaded. When compared to species above (horse, rhesus, orangutan and chimp; with the orthologous chromosome of the human chromosome 5 represented by the number inside the bar), no rearrangements were found. However, when compared to species below (rat, mouse, pig, dog, cow, opossum, and platypus) or nearby (chicken and lizard), rearrangements were identified within the region. Rearrangement breakpoints are indicated by gaps between the bars, with numbers inside each bar representing the Mb region of a chromosome (e.g., "18, 25.2-26.8" represents 25.2-26.8 Mb of chromosome 18) or a supercontig/ultra-contig/scaffold (e.g., S1,0-3.4). "Un" stands for chromosome "Unknown" in the released genome assembly. The arrow of each bar designates the sequence direction, and a dished arrow indicates that the homology to the human extends beyond 5q22.2 shown here. **B: **Human 18q21.1-q21.2 (43-50 Mb) encodes three *SMAD *genes, two *MBD *genes, *DCC*, and a number of other genes (not shown). The same as above, no rearrangements were found when compared to species shown above the human. However, when compared to the species shown below, rearrangements were found within the region and/or nearby. In addition, many sequences are missing in the orthologous chicken and lizard sites (demonstrated by large gaps in the alignment). The question mark "?" inside or below the cow bars indicates that the human-cow alignment at this region has not been completely resolved.

Through comparison of the genomic sequences of the human and other species (i.e., chimp, orangutan, rhesus macaque, marmoset, mouse, rat, guinea pig, dog, cow, opossum, platypus, chicken, lizard, xenopus, fly, worm, mosquito, honey bee, yeast, zebrafish, puffer fish, tetraodon, and ciona), we found that regions 5q22.2 and 18q21.1-q21.2 are both evolutionarily unstable (compared with an average human genomic site). Genomic rearrangements were identified in many nonhuman species (i.e., 10 out of 14 species shown in Figure 2 and all other species except xenopus listed above but not shown in Figure 2), within these sites and/or nearby regions. Hence, while genes of each region are clustered in the human genome, they are scattered on two or more distinct loci in the genome of many other species.

With several evolutionary rearrangement breakpoints identified, the human 5q22.2 region has been divided into several distinct loci in the mouse genome (Figure 2A). As a result, even though *APC *and *MCC *are less than 200 kb apart in the human genome, they are 10 Mb apart in the mouse genome (likely due to an inversion event occurring in the mouse lineage [18]). For human 18q21.1-q21.2 (Figure 2B), although the genes are clustered in the mouse genome as in the human genome, breakpoints were identified both upstream (4.5 Mb from *SMAD2*) and downstream (2.5 Mb from *MBD2*) of the region [18,19].

Other inter-species rearrangement breakpoints, shown in Figure 2, are also located at intergenic regions, and thus have not disrupted the exon/intron structure of any genes. Compared to the rest of the genome, nearly every breakpoint region contains significantly more repetitive sequences (up to 90%). For example, the one between *APC *and *MCC *contains 61% of total repeats and 26% of Alus. These percentages are significantly higher than those of the whole genome [20-22] and these repetitive sequences could have mediated non-allelic homologous recombination, facilitating inter-species rearrangements [20].

### Human 5q22.2 and 18q21.1-q21.2 regions, constantly disrupted in human CRCs, encode CRC-driver genes as well as an apparent CRC passenger gene and another questionable gene

#### *SMAD4 *versus *DCC *in 18q21.1-q21.2

The human 18q21.1-q21.2 region, constantly disrupted in human CRC [15,16], encodes the bona fide CRC gene *SMAD4 *(Figure 1), which is an essential player of the transforming growth factor-β (TGF-β) signaling pathway. This pathway controls a diverse set of cellular processes including cell proliferation, recognition, differentiation, and apoptosis during embryogenesis and in mature tissues [23,24]. *SMAD4 *has indeed been found to be deleted in human CRCs [25-27], and its driver role in tumorigenesis has been demonstrated through mouse models by knocking out either *SMAD4 *alone or both *APC *and *SMAD4 *together [28,29].

Another gene, *DCC *(*deleted in colorectal cancer *[30]), is found to be in close proximity to *SMAD4 *in the human 18q21.1-q21.2 region (Figure 2). However, unlike *SMAD4*, the mouse model studies have indicated that *DCC *is unlikely to be a CRC-driver. This is because in mice, inactive *DCC *through targeted mutagenesis [31] or spontaneous mutations (Mouse Genome Informatics, http://www.informatics.jax.org) have no effect on the intestine or on tumorigenesis within the intestine.

#### *APC *versus *MCC *in 5q22.2

The human 5q22.2 region encodes the best known CRC tumor suppressor, *APC *(*adenomatous polyposis coli*). The alteration of *APC *is the earliest event yet identified in human CRCs, and it is estimated that greater than 85% of human CRCs have somatic mutations of this gene [13]. *APC*'s tumor-suppressor role has been confirmed by various mouse models (see Mouse Genome Informatics at http://www.informatics.jax.org). *APC *encodes a multifunctional protein whose tumor suppressing function is thought to come from its ability to destabilize *β-catenin*, a key effector in the Wnt-signaling pathway [32-35]. Other roles of *APC *include mediation of intercellular adhesion, stabilization of the cytoskeleton, and possible regulation of the cell cycle and apoptosis [36]. Indeed, loss of *APC *activity has a dramatic effect on the mouse intestinal epithelium. This has been demonstrated by altered crypt/villus architecture, perturbed cell migration, increased cell proliferation and apoptosis, as well as altered gene expression [37].

Besides the bona fide tumor suppressor *APC*, 5q22.2 also encodes the gene *MCC (mutated in colorectal cancer *[38]). However, at present no evidence indicates whether *MCC *alteration contributes to human CRC development and progression or not.

#### Comparative studies with human and mouse tumors

We conducted further experimental analyses focusing on the *APC/MCC *gene pair in 5q22.2; however, we did not do so with the *SMAD4/DCC *pair in 18q21.1 for several reasons. First, unlike *DCC *where the mouse models have somewhat excluded its role in CRC [31] (see the previous section), there have been no further studies on *MCC *that directly or indirectly suggest or dispute its role in cancer since its alteration in human CRC was first reported [38]. More importantly, we were conducting comparative studies between human colorectal tumors and tumors from B6 *Apc*^Min/+ ^mice. As shown in Figure 2, unlike *SMAD4 *and *DCC *that are physically close in both genomes, *APC *and *MCC *are adjacent (< 200 kb) in the human genome but distant (> 10 Mb) in the mouse genome. Thus, the *APC/MCC *pair provided an excellent example to showcase our cross-species comparison strategy, utilizing the difference in the genomic location of orthologous genes in different species to distinguish between cancer driver and passenger alterations.

*Apc*^Min/+ ^is a well-characterized mouse model of human CRC [10,11,39-42]. The *Min *(*multiple intestinal neoplasias*) allele of *APC *was induced by ethylnitrosourea (ENU), and carries a point mutation that generates a premature stop codon (see the following section for more details) [10,11]. Studies [41,42] indicated that the remaining wild type copy of *APC *is lost through somatic recombination during intestinal tumorigenesis in B6 *Apc*^Min/+ ^mice. Thus, tumors from this model most closely resemble the majority of human sporadic CRCs where the *APC *activity is lost.

#### Bidirectional exon-resequencing

To detect sequence mutations (e.g., indels, base substitutions, and small inversions), we performed bidirectional exon-resequencing analyses on the *APC *and *MCC *genes. Along with other genes in 18q21.1-q21.2 (see the later sections), we were able to design primers for 78 out of 79 total exons. With these primers we performed PCR amplification using five pairs of colonic tumor (4-5 mm in size) and matching-normal tissue samples from two B6 *Apc*^Min/+ ^mice. We then sequenced each PCR product from both directions. Out of 3,264 total sequencing attempts (large exons required sequencing several overlapping PCR products), 3,076 successful sequences were achieved. Each successful sequence had at least 100 bp high quality bases (having a phred quality score of ≥ 20 and a base call error rate of ≤ 1%) after trimming off low quality bases. For comparison purposes, we also performed the same sequencing process on 22 pairs of human Dukes B sporadic colorectal tumors and their matching-normal tissue samples, and obtained 8,342 successful sequences from 9,360 total sequencing attempts. Thus, we achieved a sequencing success rate of 94% for the mouse samples and 89% for the human samples.

#### Sequence mutation identification

Following sequencing, we assembled the sequences for each exon using its forward and reverse sequences, based on the reference exon sequence from the published human and mouse genomes. With the assembled sequences, we performed multiple sequence alignments for the tumor/normal pairs using the CLUSTALW program. We then manually examined each alignment to ensure that the sequences were, indeed, correctly aligned (see Additional file 1 for the sequence alignments).

We identified regions in the alignment that displayed different base(s) between the paired tumor and normal sequences. To reduce false positives due to sequencing errors, we ignored bases that have a quality score of below 20. Importantly, considering that missense mutations do not necessarily change protein function as severely as nonsense or frameshift mutations, we primarily focused on base changes resulting in premature stop codons and indels within coding exons. Thus, we largely ignored base changes inside noncoding exons as well as missense mutations within coding exons.

### *APC *was disrupted in both human and mouse tumors, whereas *MCC *was disrupted in human tumors but intact in mouse tumors

#### *APC *was disrupted in both human and mouse tumors

**Figure 3:**
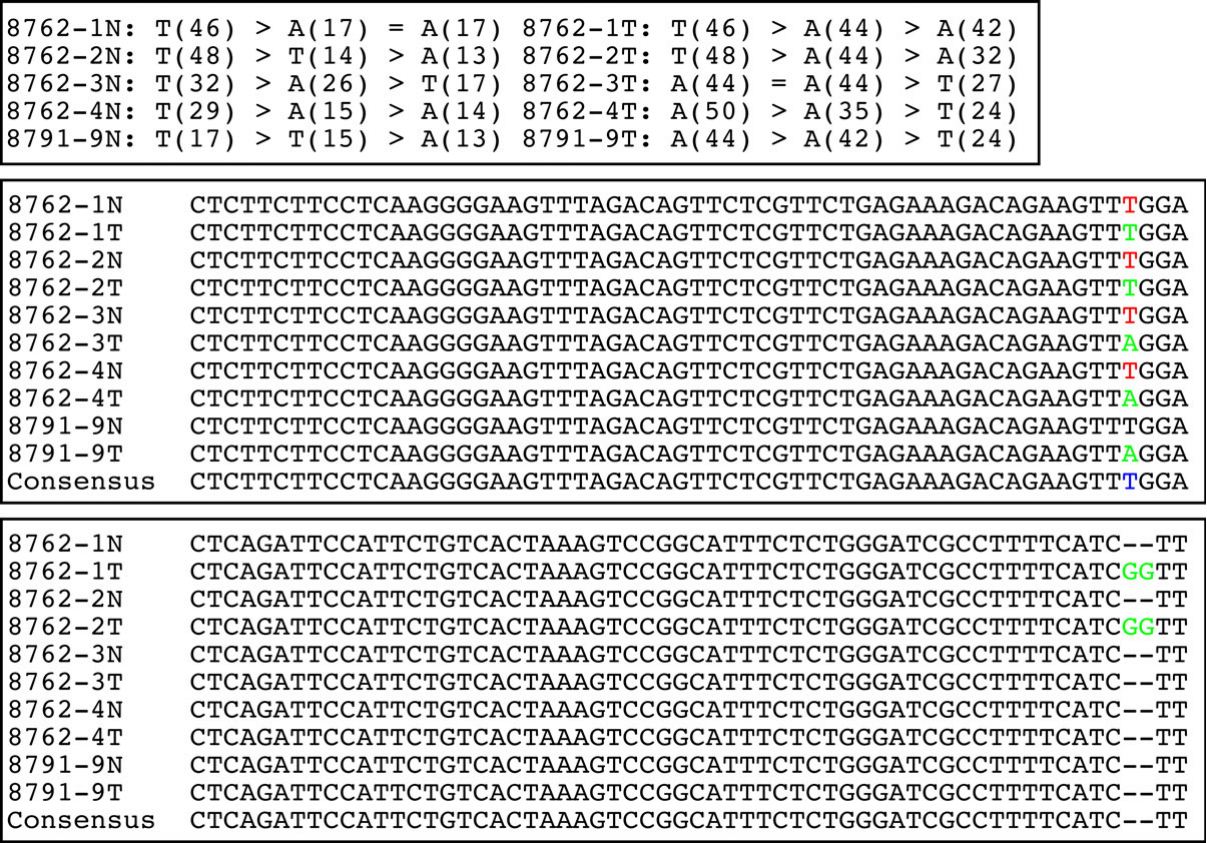
**Sequence mutations of *APC *in the tumors from B6 *Apc*^Min/+ ^mice**. **Top**: the bases and their quality score in parenthesis, e.g. T(46), are the outputs from the base-call program phred for the base 2549 of the mouse *APC *gene. The three bases next to each sample (e.g., 8762-1N), ordered by their quality score or base-call accuracy (the base call error rate equals to 10^-quality score^; thus a higher score means a more accurate base call), are from three separate sequences generated for the sample. Tumors 1-4 and their matching normal tissue samples were from mouse ID 8762, whereas tumor 5 and its matching normal sample were from mouse ID 8791 (thus, 8762-1N and 8762-1T are paired normal/tumor samples, and so on). **Middle and bottom: **The first sequence alignment is for bases 2493-2552 of mouse *APC*. Using the most accurate base (i.e., having the highest quality score) for base 2549 as indicated above, tumors 3-5 have a T- > A mutation (in red/green color) creating a premature stop codon. The remaining two tumors (8762-1T and 8762-2T) have a "GG" insertion at base 6448 of *APC*, as shown in the second sequence alignment which is for bases 6393-6450 of mouse *APC*. "Consensus" stands for the corresponding sequence obtained from the published mouse genome.

As described previously [10,11], the *Min *allele of *Apc*^Min/+ ^was created by an ENU-induced T→A point mutation at base 2,549 of the *APC *gene. This changes codon 850 from leucine-coding "UUG" to stop codon "UAG" (mouse *APC *has a total of 2,843 codons with 8,529 bases total). In our study, the base in position 2,549 was independently sequenced three times for each sample and found to be either "T" or "A" in the resulting sequences (Figure 3). This indicated that, within the same sample, some DNA molecules had "A" whereas others had "T" at this position. However, the wild type base "T" was more dominant in normal samples whereas the mutant base "A" was more dominant in tumor samples (based on the phred quality scores or the base call accuracy shown in Figure 3). These findings were consistent with the heterozygous nature of the *APC *locus, having both wild type and *Min *alleles in *Apc*^Min/+ ^mice, as well as with previous studies indicating that the wild type copy of *APC *was lost via somatic recombination in tumors [41,42]. In the two tumors (8762-1T and 8762-2T, from the same mouse) where the wild type base "T" was slightly more dominant, we identified a "GG" insertion at base 6,448, also known as codon 218 (Figure 3). This causes a frameshift for the rest of the protein, indicating that these two tumors did not retain their wild type *APC *allele either. Thus, besides somatic recombination, B6 *Apc*^Min/+ ^mice could lose their wild type *APC *copy by acquiring additional sequence mutations.

Except for these two abnormalities, we did not find any other stop codon or indel mutations for the mouse *APC *(see Additional file 1). For the human *APC*, however, we identified significantly more mutations. Stop codons and indels were found within coding exons 2-4, 6, 8, 9, 11 and/or 15 in the 22 human tumors analyzed (see Additional file 1). This is consistent with literature reports that *APC *is altered in a vast majority of the human sporadic CRCs [13], and reveals the heterogeneity of sporadic human cancers. The *APC *gene results described above demonstrated the effectiveness of our re-sequencing protocol and data-analyzing pipeline.

#### *MCC *was disrupted in human tumors but intact in mouse tumors

We sequenced each of the 17 coding exons of the mouse *MCC *gene with a total of 2,333 bases, and found no mutations in the mouse tumors (see Additional file 1) (our qPCR analysis with several of its exons did not reveal large copy number changes either). However, stop-codons were identified in several *MCC *exons in many of the human tumors studied (see Additional file 1). This is consistent with previous studies that reported *MCC *being disrupted in human CRC [38]. Thus, unlike bona fide CRC gene *APC*, *MCC *is disrupted in human tumors but intact in mouse tumors in terms of its exon sequences.

#### Confirmation of *MCC *integrity in mouse tumors by qRT-PCR

To confirm that *MCC *is intact in mouse tumors, we performed qRT-PCR analyses to examine its expression. In order to select proper normalization controls, we tested 4 genes (*β-actin, glyceraldehyde 3-phosphate dehydrogenase *or *GAPDH, 18 s ribosomal subunit*, and *β-2-microglobulin*), chosen based on a literature search. Through examination of the variation of expression in ten pairs of mouse tumor samples and matching-normal tissue samples, we designated *β-actin *and *18 s ribosomal subunit *as the normalization control genes, because no significant changes were found across the samples. We also included the *APC *gene in the study.

A total of ten pairs of adenoma and matching-normal tissue samples from several B6 *Apc*^Min/+ ^mice were analyzed. A t-test was performed to examine the differences in the normalized Ct values between paired adenoma and normal samples (Ct is the threshold cycles: the number of cycles at which the earliest measurable fluorescence signal can be detected in a qPCR assay; a higher Ct value means fewer templates). Significant statistical changes were observed for *APC *(t = 1.96 and p = 0.08), however, not for *MCC *(t = 0.71 and p = 0.5). This indicates that *APC *was altered whereas *MCC *was intact in the adenomas at the expression level, and is consistent with the resequencing results described above.

### *SMAD4 *was disrupted in both human and mouse tumors

We investigated the *SMAD4 *gene in both human and mouse tumors via sequencing and other methods for a number of reasons. First, *SMAD4 *alteration contributes to human colorectal tumorigenesis at a relatively early stage, late adenoma formation (Figure 1). Because the mouse tumors are adenomas, we would like to know if *SMAD4 *is altered or intact in these tumors. This information would provide one critical piece of evidence supporting whether or not tumorigenesis of *Apc*^Min/+ ^in mice follows similar pathways as in humans (Figure 1). Similar pathways of tumorgenesis in both species are a prerequisite for our cross-species strategy for distinguishing between cancer drivers and passengers.

Besides *SMAD4*, we also sequenced a few other genes in 18q21.1-q21.2 (Figure 2B), including methyl-CpG binding proteins *MBD1 *and *MBD2*, as controls. While we did not find stop codon or indel mutations in coding exons of the two genes in the mouse tumors, we did identify such mutations in exons 5, 6, 8, 13 of *MBD*1 (but not in any MBD2 exons) in the human tumors (see Additional file 1). These findings are consistent with the 5q22.2 sequencing analysis described above, which revealed an overall higher sequence mutation rate in the human tumors than in the mouse tumors, supporting that our sequencing analysis was run properly as well with the 18q21.1-q21.2 genes.

#### *SMAD4 *was disrupted in mouse tumors

To determine whether *SMAD*4 was disrupted or intact in the mouse tumors, we first sequenced its exons. For coding exon 7 of mouse *SMAD4*, we found that, while 60% of the matching-normal samples were sequenced successfully, all tumors failed the sequencing procedure. However, the remaining 10 coding exons (mouse *SMAD4 *has 11 coding exons with a total of 1,656 bases) were sequenced successfully with all tumor and normal samples (see Additional file 1). To determine whether the sequencing failure of coding exon 7 in the tumors was due to large sequence deletions or technical problems in our sequencing procedure, we performed further analyses as described below.

#### Confirmation by qPCR

Because qPCR is an effective strategy for detecting large deletions, we performed this analysis on a total of 10 adenomas along with their matching normal samples from several B6 *Apc*^Min/+ ^mice. We examined exon 7 (with which sequencing succeeded on most normal samples but failed on the tumors) and exon 4 (with which sequencing succeeded on all tumor/normal samples and no mutation was found) of SMAD4. We examined these along with four reference controls including exon 16 of *MCC*, exon 5 of *MBD2*, exon 10 of *MBD1*, and exon 4 of *KRAS*. These reference exons were chosen because they are all from single-copy genes in the genome and, more importantly, no large copy number changes were observed between tumor and normal samples. Our sequencing (and expression in the case of *MCC*) analyses revealed no alterations in *MCC*, *MBD2 *and *MBD1 *exons in mouse tumors (see above). For *KRAS*, we did not find any studies that reported copy number changes; most alterations identified were point mutations which would interfere less severely with the qPCR analyses, compared with large amplifications/deletions.

We noted that 8 of the 10 total tumors had a higher Ct value than their matching normal samples for exon 7 of *SMAD4*. This, however, was not observed for exon 4 of *SMAD4 *and the four reference control exons described above. To conclude these observations more quantitatively, we performed t-test analyses to examine the Ct differences between the tumors and their matching normal samples. We found that the differences were significant for *SMAD4 *exon 7 (t = 2 and p is between 0.05-0.025) but insignificant for the other exons examined (t <0.5 and p > 0.25). These results were consistent with the sequencing analyses, and indicated that *SMAD4 *exon 7 was likely deleted in the mouse tumors.

Because heterogeneity is expected for somatic mutations, we were puzzled by the apparent homogeneity observed for *SMAD4 *alteration in the mouse tumors (i.e., only exon 7 found to be disrupted). *SMAD4 *exon 7 is 51bp in size and is separated from the previous exon by an intron of 1.3 kb and from the following exon by another intron of 7.3 kb. Thus, the genomic regions surrounding *SMAD4*, exon 7, amount to 8.6 kb. These regions were found to harbor more L1 s and simple repeats with a low (< 10%) sequence divergence among the copies (i.e., younger repeats) and to be less conserved in other species (e.g., a gap of 5.8 kb for mouse and 4 kb for humans were found when aligning the corresponding sequences of this region), compared with the rest of the gene (see Additional file 1). We hypothesize that deletions of exon 7 could have occurred via homologous recombination between the two highly identical AT-rich repeats inside its two flanking introns (see Additional file 1). Hence, the apparent homogeneity could have arisen from the fact that sequences around exon 7 are intrinsically more unstable, compared with those of other parts of the gene. A somewhat similar situation has been reported for the *BRCA1 *gene locus, of which the structural instability revealed by evolutionary analyses may mostly originate from Alu-mediated rearrangement [43].

Our hypothesis was partially supported by the observation of "subtle" chromosome instability (CIN) in the mouse tumor genomes. By analyzing several mouse tumors with Roche NimbleGen's 384 K oligonucleotide comparative genomic hybridization (CGH) arrays, we found that ~4% of the genomic sequences of the tumors were amplified/deleted, compared to 1% when hybridizing a normal sample against another normal sample. This is consistent with a recent study that observed misoriented spindles and misaligned chromosomes associated with tetraploid genotypes in dividing crypt cells within the small intestines of *APC*^*Min/+ *^mice [44]. However, we must emphasize the word "subtle" for the observed mouse CIN because, for the human tumors displaying CIN, the amplified/deleted sequences amount to >10% of the genome. This, perhaps, explains why other studies did not find CIN in *Apc*^Min/+ ^tumors [45]. The reason that "subtle" CIN was detected here is, most likely, due to the high resolution of the oligo arrays being used (one probe every 5-6 kb across the genome), and, as far as we know, we are the only group that has used such high density arrays on these mouse tumors.

With the Ct difference between the tumors and their matching normal samples ranging from -0.1 to 2, the 10 tumors used in qPCR analyses showed more heterogeneity than the 5 tumors used in the sequencing analysis for *SMAD4 *exon7 (all tumors failed the sequencing process). One likely reason is that these 5 tumors were considerably larger than those used in qPCR analyses (4-5 mm versus 1-2 mm in size). Therefore, they were at later tumor progression stages, where more extensive genomic instability develops and more genomic abnormalities accumulate.

#### *SMAD4 *in human tumors

A high sequencing failure rate was also found in the human homologue of the mouse *SMAD4 *coding exon 7. There were also disruptions in a number of other coding exons (see Additional file 1), indicating that *SMAD4 *was also altered in the human tumors, consistent with previous studies [25-27].

### *P53 *also altered in mouse tumors

Besides genes of 5q22.2 and 18q21.1-q21.2 described above, we also investigated genes with high mutation prevalence (i.e., *P53*, *KRAS*, and *PIK3CA*), as well as those with intermediate-low mutation prevalence (i.e., *SMAD2 *and *SMAD3*) in human CRC [46]. This would provide more evidence to evaluate the molecular similarities of tumorigenesis between human CRC and the *Apc*^Min/+ ^model.

We conducted qRT-PCR analysis on these genes along with two reference genes *β-actin *and *GAPDH*, with 12 matched colon adenomas and normal tissue samples from several *Apc*^Min/+ ^mice. After normalization with *β-actin*, we performed t-tests to determine if the Ct difference between the tumors and normal samples is significant for each gene. We found that *P53 *was significantly altered in these tumors (t = -4.8; p < 0.0001). However, we did not detect significant changes for the other genes (*KRAS*, *PIK3CA, SMAD2*, and *SMAD3*), although their t-values are >10 times larger than that of *GAPDH *(see Table s1 in Additional file 1). Hence, this analysis indicates that *P53 *was also altered in the mouse tumors, adding another piece of evidence supporting the molecular similarities between human CRC and the mouse model. For the other genes, further analysis (e.g., sequencing) are clearly needed to determine if they are intact or disrupted in the mouse tumors.

## Discussion

**Figure 4:**
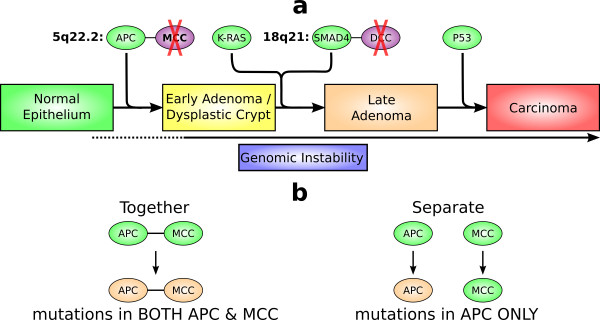
**Our study indicates that *MCC *alteration is unlikely to be a driver of colorectal tumorigenesis at early stages (top)**. The bottom illustrates our hypothesis that alteration of *MCC *in human CRCs occurs as a result of its proximity to *APC *in the human genome (~200 kb apart). However, because *MCC *is distant from *APC *(~10 Mb apart) in the mouse genome, agents that caused *APC *alternations have no effect on *MCC*; thus, like other genes without causative roles in tumorigenesis, *MCC *remains intact in the mouse tumors.

The ability to distinguish between cancer driver and passenger alterations has been a central aim of cancer research. To achieve this, we are developing a cross-species comparison strategy by taking advantage of the difference in the genomic location of orthologous genes between the human and other mammals. To showcase this strategy, we conducted a pilot study focusing on genes *APC *and *MCC*, which are adjacent in 5q22.2 of the human genome but distant in the mouse genome (Figure 2). We found that, consistent with literature reports, both genes were disrupted in human colorectal tumors. However, in the mouse tumors, only bona fide CRC gene *APC *was disrupted whereas *MCC *appeared to be intact. This indicates that *MCC *alteration may not be a driver (but rather a passenger) of colorectal tumorigenesis, and we hypothesize that its disruption in human CRCs could merely be a result of its close proximity to *APC *in the human genome (Figures 2 &4), as rationalized as follows. When mutagens act on the *APC *locus causing *APC *mutations, the neighboring *MCC *gene is accidently exposed to the mutagens (consistent with this, we also found a significant amount of nonsense mutations and indels in another neighboring gene *DP1 *or *deleted in polyposis 1*; see Additional file 1). Because *MCC *is 10 Mb away from *APC *in the mouse genome (Figure 2), agents that cause *APC *alteration may essentially have no effect on *MCC*. Hence, *MCC *could simply be like other background genes with no causative roles (Figure 4), and thus no enrichment of *MCC *alteration was observed in the mouse tumors. Perhaps a more familiar analogous example of this situation would be: a passenger was injured in a car accident that was caused by the driver of the car (*MCC *vs. *APC *in the human tumors); however, if the passenger had been sitting in a different car, he/she would, most likely, have been injury-free (*MCC *vs. *APC *in the mouse tumors).

This pilot study demonstrates the promise of using this cross-species comparative strategy to identify cancer driver alterations. Nearly 10,000 loci have been found to be disrupted in human colonic polyps [47]; clearly, it would be useful to know which disruptions are colorectal tumorigenesis-drivers and which ones are not. Several groups, including us, have built a high resolution synteny/rearrangement map between the human and mouse genomes [8,9,18], and identified ~400 inter-species rearrangements. Thus, there are many places like the *APC/MCC *locus in the human genome. Provided that the B6 *Apc*^Min/+ ^model is clearly demonstrated to share similar cancer progression pathways as human CRCs (discussed below), expanding this pilot project to the entire genome would potentially identify many targets like *MCC*, facilitating bona fide CRC gene discovery.

The disruption of *SMAD4 *in mouse tumors raises the possibility that B6 *Apc*^Min/+ ^mice might share a similar genetic pathway of tumorigenesis as human CRC shown in Figure 1[12,13], especially considering that *SMAD4 *is distantly located from the ENU-induced *Apc*^*Min/+ *^locus (Figure 2). This is further supported by our observation that the expression of *P53 *is also significantly altered in mouse tumors. Consistent with these, several recent studies reported that a subset of the genes, with expression altered in *Apc*^Min/+ ^adenomas, were confirmed to be changed in human CRCs as well [40,48]. In addition, our high density aCGH array analysis found subtle CIN in the genome of the mouse adenomas, which is consistent with the human colorectal tumorigenesis model where CIN is an early event (Figure 1). Of course, a significant amount of work is needed to conclude that *APC*^*Min/+ *^tumorigenesis indeed share the similar pathway as human CRC as shown in Figure 1, such as investigating other contributors such as *KRAS *gene alteration.

Besides conducting further studies with B6 *Apc*^Min/+^, other mouse models should also be explored. This is especially needed when comparing more genetically homogeneous gene-knockout mouse models to more genetically heterogeneous sporadic human CRCs. The *APC*^*Min/+ *^model may only represent a subset of sporadic human CRCs. This could be improved by studying other mouse models, such as *SMAD4*-knockout mouse models [28,29] and those described in a recent publication (azoxymethane or AOM, *Smad3*^-/-^, and *Tgfb1*^-/- ^*Rag2*^-/-^) [48]. Importantly, tumors from *APC*^*Min/+ *^are mostly adenomas, and obviously *APC*^*Min/+*^cannot represent late stage human CRCs. Other models, such as the one described by Hinoi *et al. *[49] that aims to model transition from adenoma to adenocarinoma, should be investigated. Of course, sporadic cancers from animal models would be the best representation of sporadic human cancers [50], but sporadic cancers are rare in mice and difficult to obtain in other species. However, essentially an unlimited amount of tumors could be accessed with the genetically modified mouse models, which greatly facilitates the studies.

Unlike strategies such as gene knockout mouse models, our approach does not provide direct evidence to determine whether a gene alteration is a cancer driver or passenger. However, this approach is much more cost- and time-effective, as performing large scale analyses, such as microarray or sequencing studies, could allow all the genes and the entire genome of an organism to be examined in a single experiment. Our approach could quickly narrow down the list of cancer-driver gene alteration candidates, which then can be verified by direct approaches, such as gene knockout mouse models.

## Conclusions

We conducted a pilot study to demonstrate the effectiveness of a cross-species comparison strategy to distinguish between cancer driver and passenger alteration candidates, by utilizing the difference in the genomic location of orthologous genes and genomic loci between the human and other mammals. This pilot study focused on the human 5q22.2 and 18q21.1-q21.2 regions, which are frequently disrupted in human CRCs. They also encode bona fide cancer genes (e.g., *APC, SMAD4*), an apparent passenger gene, *DCC*, and genes that are disrupted in human CRCs but whose roles in cancer remain unclear (e.g., *MCC*). Both regions are evolutionarily unstable. As a result, *APC *and *MCC *are adjacent in the human genome but are distant in the mouse genome. By studying the same orthologous genes in colon tumors from humans and the CRC mouse model B6 *Apc*^*Min/+*^, we found that bona fide cancer genes *APC *and *SMAD4 *were disrupted in both species. However, the questionable cancer gene *MCC *appeared to be intact in the mouse tumors, unlike in the human tumors. This indicates that *MCC *may not be a driver of colorectal tumorigenesis, and that its alteration in human CRCs could be due to its close proximity to *APC *in the human genome (Figures 2 &4). This pilot study demonstrates the promise of using this cross-species comparative genomics and oncology strategy to identify cancer-causative alterations. Once the B6 *Apc*^Min/+ ^model is clearly demonstrated to share similar tumorigenesis pathways as in humans CRCs (Figure 1), expanding this pilot project to the entire genome would potentially identify many targets like *MCC*, facilitating cancer driver gene alteration discovery.

## Competing interests

The authors declare that they have no competing interests.

## Authors' contributions

XJ and JT analyzed the sequence data. RH and MP provided the mouse tumors and associated pathological information. DB and SF did the sequencing work. CV and TJ provided the human DNA samples and associated pathological/clinical information. SZ participated in conducting the qPCR and qRT-PCR analyses, designed the study, and wrote the manuscript. All authors contributed to the manuscript editing. All authors read and approved the final manuscript.

## Pre-publication history

The pre-publication history for this paper can be accessed here:

http://www.biomedcentral.com/1471-2407/10/426/prepub

## Supplementary Material

Additional file 1 **Supporting Material**. This file provides additional analyses and information to support the conclusions described in the main text. Click here for file
