# Supplementary material for: Distinguishing between cancer driver and passenger gene alteration candidates via cross-species comparison: a pilot study
Source: BMC Cancer. 2010 Aug 13;10:426. doi: 10.1186/1471-2407-10-426 (PMC2927548; doi:10.1186/1471-2407-10-426)
Supplement: Additional file 1 — Supporting Material. This file provides additional analyses and information to support the conclusions described in the main text. [file 1471-2407-10-426-S1.DOC]

**Supporting Material**

**The mouse exon-resequencing analyses including the sequence alignment for all the genes studied can be found at:** [http://rich.yunda.org/uga/science/cclone/06032101/mouse/processed/05.convert/index.html](http://csbl.bmb.uga.edu/~jix/science/cclone/06032101/mouse/processed/05.convert/index.html)

**The human exon-resequencing analyses including the sequence alignment for all the genes studied can be found at:** [http://rich.yunda.org/uga/science/cclone/06032101/human/HumanColonSample1st2Batch/index.html](http://csbl.bmb.uga.edu/~jix/science/cclone/06032101/human/HumanColonSample1st2Batch/index.html)

<http://rich.yunda.org/uga/science/cclone/06032101/human/HumanColonSample2ndBatch/index.html>

**Besides the two abnormalities described in text, no other sequence mutations were identified in the mouse *APC* coding exons (CDSs),** as demonstrated by the sequence alignment shown at: [http://rich.yunda.org/uga/science/cclone/06032101/mouse/processed/05.convert/mAPC/mAPC.step8.index.html](http://csbl.bmb.uga.edu/~jix/science/cclone/06032101/mouse/processed/05.convert/mAPC/mAPC.step8.index.html)

***APC* was found to be disrupted in human tumors,** as demonstrated by the sequence alignment shown at: [http://rich.yunda.org/uga/science/cclone/06032101/human/HumanColonSample1st2Batch/APC/APC.step8.index.html](http://csbl.bmb.uga.edu/~jix/science/cclone/06032101/human/HumanColonSample1st2Batch/APC/APC.step8.index.html)

**No mutations was found in 17 coding exons or CDSs of *MCC* in mouse tumors,** as demonstrated by the sequence alignment shown at: [http://rich.yunda.org/uga/science/cclone/06032101/mouse/processed/05.convert/mMCC/mMCC.step8.index.html](http://csbl.bmb.uga.edu/~jix/science/cclone/06032101/mouse/processed/05.convert/mMCC/mMCC.step8.index.html)

***MCC* was found to be disrupted in human tumors,** as demonstrated by the sequence alignment shown at: <http://rich.yunda.org/uga/science/cclone/06032101/human/HumanColonSample2ndBatch/MCC/MCC.step8.index.html>

**No sequence mutations were found in the CDSs of mouse *SMAD2, MBD1,* and *MBD2***, as demonstrated by the sequence alignment respectively shown below:

[http://rich.yunda.org/uga/science/cclone/06032101/mouse/processed/05.convert/mMAD2/mMAD2.step8.index.html](http://csbl.bmb.uga.edu/~jix/science/cclone/06032101/mouse/processed/05.convert/mMAD2/mMAD2.step8.index.html)

[http://rich.yunda.org/uga/science/cclone/06032101/mouse/processed/05.convert/mMBD1/mMBD1.step8.index.html](http://csbl.bmb.uga.edu/~jix/science/cclone/06032101/mouse/processed/05.convert/mMBD1/mMBD1.step8.index.html)

[http://rich.yunda.org/uga/science/cclone/06032101/mouse/processed/05.convert/mMBD2/mMBD2.step8.index.html](http://csbl.bmb.uga.edu/~jix/science/cclone/06032101/mouse/processed/05.convert/mMBD2/mMBD2.step8.index.html)

**Small indels were identified in exon 8 (CDS7) of *MBD*1 in the human tumors**, as demonstrated by the sequence alignment shown at: [http://rich.yunda.org/uga/science/cclone/06032101/human/HumanColonSample1st2Batch/MBD1/MBD1_EXON8.html](http://csbl.bmb.uga.edu/~jix/science/cclone/06032101/human/HumanColonSample1st2Batch/MBD1/MBD1_EXON8.html)

**CDS7 (exon 8) of *SMAD4* was found to be disrupted in a majority of the mouse tumors, while other coding exons were found to be intact**, as demonstrated by the sequence alignment shown at: [http://rich.yunda.org/uga/science/cclone/06032101/mouse/processed/05.convert/mMAD4/mMAD4.step8.index.html](http://csbl.bmb.uga.edu/~jix/science/cclone/06032101/mouse/processed/05.convert/mMAD4/mMAD4.step8.index.html)

***SMAD4* was found to be disrupted in the human tumors,** as demonstrated by the sequence alignment shown at: [http://rich.yunda.org/uga/science/cclone/06032101/human/HumanColonSample1st2Batch/MADH4/MADH4.step8.index.html](http://csbl.bmb.uga.edu/~jix/science/cclone/06032101/human/HumanColonSample1st2Batch/MADH4/MADH4.step8.index.html)

**Repeat content and sequence conservation information can be found at UCSC genome site at** [**http://www.genome.ucsc.edu**](http://www.genome.ucsc.edu/). The following are for the mouse July 2007 assembly (mm9) version.

- Left flanking intron of exon 7 of SMAD4 (chr18:73,816,900-73,818,221) contains an AT-rich simple repeat of 8.1% divergence at chr18:73817126-73817187.
- Right flanking intron of exon 7 of SMAD4 (chr18:73809564-73816848) contains another AT-rich simple repeat of 8.8% divergence at chr18:73810380-73810436, a young L1 of 8.8% divergence at chr18:73812478-73812579, a (CA)n repeat of 0% divergence at chr18:73812589-73812636, and a (TG)n repeat of 18% divergence at chr18:73812722-73812792.

Table s1. T-test results on gene expression alteration

| Gene | *P53* | *SMAD3* | *SMAD2* | *PIK3CA* | *KRAS* | *GAPDH* |
| --- | --- | --- | --- | --- | --- | --- |
| t-value | -4.80 | -0.99 | 0.91 | 0.90 | -0.21 | 0.02 |
| p-value | <0.001 | p<0.2 | p<0.2 | p<0.2 |  |  |
